# Enhancing the Innovation Ecosystem: Overcoming Challenges to Introducing Information-Driven Technologies in Health Care

**DOI:** 10.2196/56836

**Published:** 2025-03-24

**Authors:** Julie Reed, Petra Svedberg, Jens Nygren

**Affiliations:** 1 School of Health and Welfare Halmstad University Halmstad Sweden

**Keywords:** artificial intelligence, ecosystem, health care, implementation, technology adoption, improvement, complex-systems

## Abstract

As health care demands rise and resources remain constrained, optimizing health care systems has become critical. Information-driven technologies, such as data analytics and artificial intelligence (AI), offer significant potential to inform and enhance health care delivery at various levels. However, a persistent gap exists between the promise of these technologies and their implementation in routine practice. In this paper, we propose that fragmentation of the innovation ecosystem is behind the failure of new information-driven technologies to be taken up into practice and that these goals can be achieved by increasing the cohesion of the ecosystem. Drawing on our experiences and published literature, we explore five challenges that underlie current ecosystem fragmentation: (1) technology developers often focus narrowly on perfecting the technical specifications of products without sufficiently considering the broader ecosystem in which these innovations will operate; (2) lessons from academic studies on technology implementation are underused, and existing knowledge is not being built upon; (3) the perspectives of healthcare professionals and organizations are frequently overlooked, resulting in misalignment between technology developments and health care needs; (4) ecosystem members lack incentives to collaborate, leading to strong individual efforts but collective ecosystem failure; and (5) investment in enhancing cohesion between ecosystem members is insufficient, with limited recognition of the time and effort required to build effective collaborations. To address these challenges, we propose a series of recommendations: adopting a wide-lens perspective on the ecosystem; developing a shared-value proposition; fostering ecosystem leadership; and promoting local ownership of ecosystem investigation and enhancement. We conclude by proposing practical steps for ecosystem members to self-assess, diagnose, and improve collaboration and knowledge sharing. The recommendations presented in this paper are intended to be broadly applicable across various types of innovation and improvement efforts in diverse ecosystems.

## Introduction

### Focus and Aim of Viewpoint

Despite a sizeable increase in the development of new information-driven technologies in health care their uptake and impact in routine practice remains limited. This viewpoint argues that this failure stems from fragmentation of the innovation ecosystem which is driving a lack of synergist working between ecosystem members. This includes all stakeholders who have a role to play in conceptualizing, designing, developing, studying, implementing, regulating, and using such technologies. We propose that in order to realize the potential of new technologies in health care we need to improve how the innovation ecosystem is working. This is especially true as we tackle bigger and more impactful innovations that require a greater degree of coordinated action.

Drawing on our experiences and published literature we explore the challenge of current ecosystem fragmentation and the underlying system drivers that make collaboration and synergistic working so difficult to achieve. We make a series of recommendations as to how these challenges can be addressed including the need to adopt a wide-lens perspective on the ecosystem and develop a shared-value proposition, a call for ecosystem leadership, and the need for local ownership of ecosystem investigation and enhancement. We finish by proposing practical steps ecosystem members can take to self-assess, diagnose, and enhance how they are working and learning together.

The recommendations presented in this paper are intended to be broadly applicable across various types of innovation and improvement efforts in diverse ecosystems.

### Background

The current strain on the health care system highlights the necessity for improving quality of care, with a specific emphasis on promoting effectiveness and efficiency in a manner that best serves the needs of patients and populations while promoting the well-being of health care staff members. It is therefore imperative to address these challenges and implement strategies that not only enhance health care delivery but also ensure the sustainability of health care systems.

Within this backdrop, the advent of cutting-edge technologies, encompassing advancements in data analytics, computational capabilities, and artificial intelligence (AI), provides an opportunity to inform the provision of health care [1,2]. Information-driven technologies in health care encompass systems and tools that rely on the collection, integration, and analysis of vast volumes of data to support and enhance clinical and operational decision-making [1,2]. These technologies are fundamentally dependent on the availability of high-quality, diverse, and interoperable datasets, as their effectiveness is directly tied to the comprehensiveness and accuracy of the underlying information. Central to their functionality are advanced analytics, particularly AI techniques, which enable the processing of complex and multidimensional datasets to identify patterns, generate predictions, and provide actionable insights. For example, AI can detect subtle anomalies in diagnostic imaging, predict disease progression based on longitudinal patient data, or optimize care pathways by analyzing historical treatment outcomes. By automating these sophisticated analyses, information-driven technologies could facilitate precision medicine, improve the allocation of health care resources, and reduce inefficiencies. However, despite the potential benefits of integrating information-driven technologies into health care systems, there is limited evidence of their successful uptake into routine health care practice [3,4].

Drawing inspiration from Ron Adner’s book “The Wide Lens” we propose that the limited adoption of information-driven technologies in health care stems from technology developers focusing too narrowly on their individual products without considering the entire ecosystem needed for success. Adner [5,6] argues that any innovator requires collaboration with other co-innovators, who develop complementary solutions to make the focal innovation viable, and with adoption chain partners who must adopt or support the innovation before it can reach the end user. This wide-lens perspective emphasizes that the success of an innovation depends not only on the product itself but also on external groups. To minimize the risk of failure, innovators need to identify and align with the interests of these ecosystem players and proactively manage their dependencies.

For example, Amazon’s success with the Kindle e-reader contrasts with Sony’s earlier failure to launch a similar product. Adner explains that Sony’s focus on perfecting the device’s technical specifications overlooked the broader ecosystem requirements. Without securing coinnovators like publishers to provide e-books, a smooth platform for adoption chain partners such as booksellers to distribute them, and a seamless experience for buyers (the adopters), the product could not deliver the full value customers needed, leading to its failure. To succeed, Amazon took on a key leadership role by motivating and coordinating contributions from a diverse range of ecosystem members, ensuring that all necessary innovations and activities were in place to support their new product.

We believe that such ecosystem leadership and coordination is currently lacking in health care innovation, and the failure to consider how innovations work within the broader health care ecosystem is a key reason why new information-driven technologies struggle to gain traction.

In this viewpoint, we explore challenges of fragmentation that are specific to the health care innovation ecosystem and make a series of recommendations to drive ecosystem cohesion and success.

## The Challenge: Ecosystem Fragmentation

### Overview

To consider the fragmentation of the health care innovation ecosystem we need to explore the relationships between different ecosystem members. The wider ecosystem of groups and organizations contributing to the health care innovation ecosystem is vast, comprising of diverse stakeholders and actors from different organizations, as well as professionals and experts from a broad spectrum of disciplines [7]. This includes health care organizations responsible for planning and delivery of care, industry organizations engaged in technology development and commercialization, academics who can contribute expert knowledge and methodological insights, and community, charitable, and voluntary organizations who play a variety of support roles to the well-being of patients and the wider population, and government and regulatory organizations who legislate and regulate activities [8,9].

For the purpose of this viewpoint, we have chosen to focus on the relationships between three main groups of ecosystem members to illustrate the challenges of ecosystem fragmentation: (1) the innovators who develop new technologies, (2) researchers who study the use and implementation of technologies, and (3) health care professionals who are the intended adopters and users of the technologies in practice. We recognize that in practice exploration of ecosystem relationships would require expansion to include all relevant members (recommendations).

### Challenge #1: Not Looking Over the Fence—a Narrow Focus on Innovation Pipelines

Technology developers often adopt a narrow lens, focusing exclusively on their own innovation pipelines while neglecting the broader ecosystem in which their innovations must operate. This tendency is particularly evident in the development of information-driven technologies in health care, where the primary emphasis is often on data availability and technological functionality [10]. Critical issues such as usability, implementation in routine care, and the interdependence with existing health care practices and processes are frequently treated as afterthoughts, only considered after the proof-of-concept stages [3,4].

**Figure 1:**
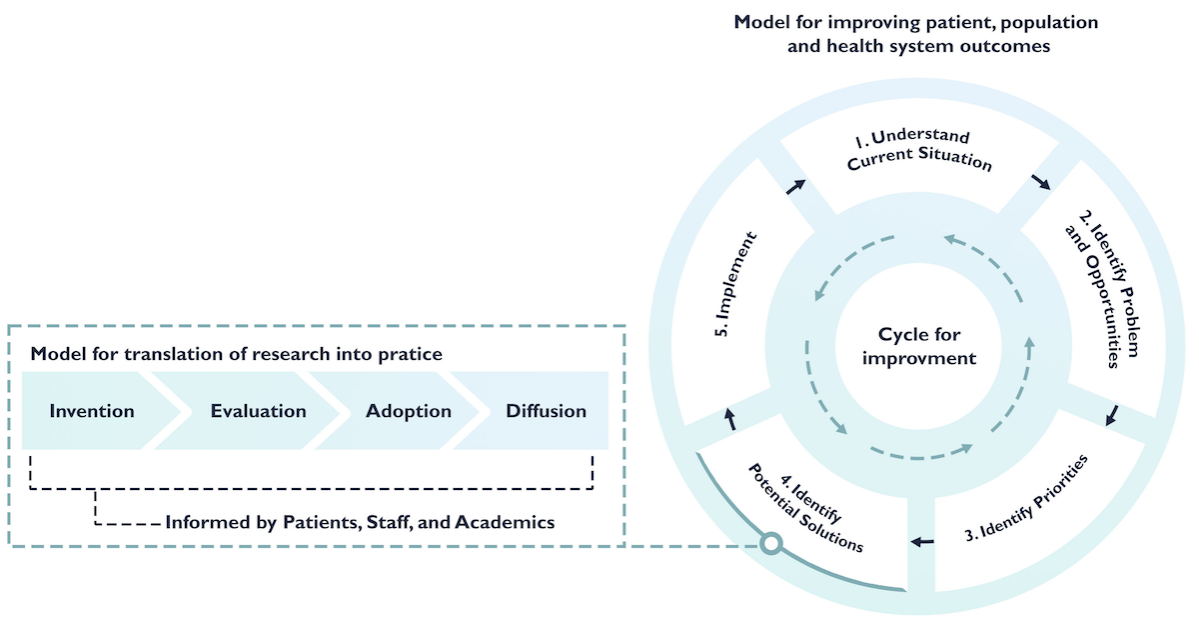
Models for conceptualizing the innovation process: Traditional linear model of innovation-led development (left) needs to be reconceptualized as part of a more holistic cycle for improvement (right), which focuses on achieving system aims and goals and increases requirements for collaborative working between innovators and practitioners.

Traditional conceptualizations of the innovation process exacerbate this narrow focus. The linear innovation pipeline model (Figure 1) [11] assumes a straightforward handoff between developers and adopters after invention and evaluation, or proof-of-concept, as it is often termed in technology development. This perspective perpetuates the misconception that bridging the gap between vision and practice, often referred to as “the last mile” [12], is primarily the adopters’ responsibility. However, such an approach overlooks the critical dependencies and coinnovations required for successful integration into health care systems [13].

This narrow focus on technological development often results in solutions that fail to meet the real-world needs of health care staff members or patients. Technologies that ignore the routines, constraints, and practicalities of health care delivery risk being fundamentally misaligned with the requirements of adopters. Even well-designed technologies can falter without understanding the broader ecosystem changes needed to support their use.

From an innovator’s perspective, this pipeline model may seem logical, it allows a focus on technical excellence within one’s domain of expertise. However, it risks creating solutions based on overly simplistic notions of how health care systems function, ignoring the complexities and constraints of real-world applications. For example, health care professionals often face an overabundance of information, not a lack of it. While many information-driven technologies aim to provide more precise or accurate data, a more pressing challenge is navigating the “noise” of overwhelming information, knowing how to act on it, and having the resources and authority to do so. Thus, technology developers need to spend time understanding these challenges or the broader concerns of other ecosystem members.

### Challenge #2: Not Building on What we Know—Lessons From the Study of Technology Use and Implementation Are Underused

The limitations of the “narrow lens” of product development and the failure to consider the wider context of technology deployment are well-documented. For decades, health care research has highlighted the challenges of diffusing and adopting innovations in practice (eg, NASSS [Non-adoption, abandonment, scale-up, spread, and sustainability] framework) [14,15]. Studies of technology use, such as Normalization Process Theory [16] and sociomaterial approaches [17] have repeatedly emphasized the importance of understanding the interaction between technology, its users, and the wider context of deployment, echoing Adner’s findings [5]. While much of this research is not specific to information-driven technologies, its insights into the enablers and barriers to innovation uptake are both relevant and generalizable.

Despite this extensive body of knowledge, it has not significantly influenced the behaviors and practices of technology developers or their collaborations with other ecosystem members. In most industries, such research would be a cornerstone of product development; yet in health care, even with substantial investment in research, its findings often fail to be acted upon.

Awareness of these studies is low among tech developers. For example, a recent systematic review found that implementation theories were rarely applied in the or implementation of AI technologies for health care [3]. Even within academic disciplines that study health care technology and innovation, such as implementation science and sociomaterial studies, there is recognition of the limited practical impact of their findings [18-20].

The lack of uptake of such knowledge is not solely the fault of technology developers. Multiple complex factors contribute to this fragmentation. Studies often report qualitative findings, embedded in psychological, sociological, or organizational perspectives, and presented as nuanced theories written in discipline-specific language. This can feel alien and inaccessible to those outside these academic fields, including technology developers. The diversity of disciplines conducting these studies, each with distinct priorities, perspectives, and terminologies, further complicates understanding. Even for experts in health care research, navigating these different theoretical perspectives can be daunting.

In addition, many studies stop at describing problems without providing actionable guidance for solutions. While technology developers may focus narrowly on their products, researchers often focus narrowly on publishing theories, with less attention to their practical application. This leaves developers without clear, actionable steps to implement research findings in their work. Bridging the gap between research and practice requires collaborative approaches that translate theoretical insights into practical guidance, tailored to the needs of technology developers and other ecosystem members.

### Challenge #3: Not Coordinating with Health Care Needs—Overlooking the Perspectives of Health Care Professionals and Organizations

Research on health care innovation consistently highlights that the success of new technologies hinges on their fit with the context in which they are introduced [21]. Yet, the perspectives of health care professionals are often insufficiently considered during development.

Health care professionals play two critical roles in this process. As adoption-chain partners, they ensure innovations integrate into practice and are used appropriately. For example, a new technology would need to be integrated into existing workflows, staff members trained on how to use it, and maintenance support systems in place. Without this work to actively support technology adoption the innovation will not be successfully implemented. As coinnovators, they create complementary innovations necessary for technologies to achieve their full value. For example, a new technology may be very powerful in identifying patients at risk of having a particular health condition, but would require coinnovation to identify how to reach out to these patients to notify them of this risk, and how to treat them. Without these coinnovations, the technology would not be realizing its value to the health care ecosystem.

Both adoption-chain partners and coinnovators should be engaged from the outset of any technology development to work collaboratively to align incentives, address technical and operational challenges, and mitigate potential conflicts. While there is some recognition of the need to engage adoption-chain partners, this process is often superficial and assumes that health care professionals can easily provide the necessary insights. In reality, this assumption is flawed. Health care systems are extraordinarily complex, with knowledge distributed across many individuals and much of it tacit. Consequently, developers and researchers often base their work on incomplete understandings of “work as imagined” rather than “work as it is.” Obtaining reliable insights into workflows, practices, and behaviors requires time, expertise, and resources that neither technology developers nor health care professionals typically have. This disconnect often results in poorly defined briefs and high-specification products that fail to meet the needs of the system, frustrating both developers and users.

Coinnovation is rarely prioritized. Many developers focus narrowly on developing accurate, effective tools, assuming these will inherently provide value. However, the true value of any technology lies not in the information it provides but in its ability to improve clinical practices and patient outcomes. Achieving this requires additional innovations, such as modifying dependent processes and practices, and changing professional behaviors, tasks that are often left to health care organizations [22]. These organizations often lack the resources, expertise, or capacity to manage such work, particularly given the competing demands of delivering care and supporting staff members well-being in under-resourced and high-stress environments. For them, technology is not the focal point but a potential enabler of better care.

Adding to these challenges, frontline teams are frequently overwhelmed by the sheer volume and variety of new interventions they are expected to adopt, often with little consideration of how these initiatives interact or how unresolved challenges might be exacerbated by new technologies [19]. At present, there is insufficient coordination within the health care ecosystem to address these challenges. Innovators often view technology as the central actor, whereas health care teams see it as a supportive tool for achieving their goals. This misalignment leads to tension and hinders the effective uptake of new innovations.

### Challenge #4: Not Incentivizing People to Work Together—Individually People Are Doing Good Jobs, But Collectively the Ecosystem Is Failing

In the current health innovation ecosystem, individuals excel in their specific roles: technology developers create apps and algorithms, health researchers produce theories and publications, and health care professionals deliver services. However, the ecosystem often fails collectively because members are incentivized to focus on predetermined end points within their disciplines rather than working collaboratively across boundaries. No one is tasked with bridging the gaps between these roles or ensuring that end products are effectively integrated.

This fragmentation fosters a false sense of completion, where apps are handed off to health care teams or theories to developers without accountability for their practical impact. Crucial feedback loops, such as whether the app or theory is used and helpful, are neglected. Without this cross-disciplinary exchange, the ecosystem remains siloed, disconnected, and unable to adapt to the needs and constraints of its members.

A notable example of ecosystem failure due to the fragmentation of responsibilities, where tasks are divided into manageable parts but lack a comprehensive overview and accountability, is the collapse of the Minneapolis I-35W Bridge on August 1, 2007. Investigation into the disaster identified that all individual contractors including the original design firm, transportation officials, inspection teams, and maintenance crew had fully met their individual responsibilities, and as such could not be blamed for the disaster. However, collectively they had failed due to the fragmentation of responsibilities between multiple entities which resulted in a combination of design flaws, a lack of adaptation to the incremental increases in the bridge’s weight over time, and decades of inadequate maintenance. In health care innovation, fragmentation results in frustration: developers lament the lack of adoption [23,24], researchers feel their insights are underused [25], and clinicians face an unrelenting influx of innovations that fail to address their real challenges [26]. Despite these frustrations, each group often blames other ecosystem members or assumes the problem lies outside their responsibility.

Career pathways exacerbate this issue. Academic success depends on publishing prestigious theories, not translating them into practice. Developers are rewarded for producing tools, not ensuring their usability. Clinicians focus on delivering care, not system-wide coordination. These narrow incentives discourage collaborative work and maintain the status quo. To step outside these norms often requires entrepreneurialism whilst risking personal career security.

The result is not that people are doing their jobs poorly but that they are working to end points too narrow to achieve broader ecosystem success. Effective ecosystem collaboration requires questioning assumptions, addressing gaps, and building strong relationships. Members must challenge briefs, clarify expectations, and engage in active feedback loops. A cohesive system depends on recognizing and valuing the interdependence of roles and the shared responsibility for collective success.

### Challenge #5: Not Investing in Working Across Boundaries—Lack of Recognition of the Time and Effort Required

The diversity of ecosystem members is a strength, yet it also presents a significant, often unacknowledged challenge. Different scopes of interests, priorities, expertise, and practices can make it difficult for groups to share knowledge and work together effectively. It is much easier to learn from others within your own field, where shared language and mindsets foster understanding [27]. However, this specialized language can create barriers when trying to communicate with professionals from different fields [28]. At present little time and effort is invested, and few career pathway opportunities are provided to develop proficiency in navigating these boundaries or building shared understanding.

Communication efforts between ecosystem members often consist of one-way transfers of information, where one group assumes the role of “teaching” and the other as the “learner.” These approaches fall short in 2 critical ways: they fail to consider the effort required to change behaviors and work processes, and they overlook the importance of 2-way, iterative learning [29]. Effective collaboration should focus on understanding the implications of each group’s work for the others.

For instance, health research may highlight the need for technology developers to better understand day-to-day health care practices. However, developers typically focus on desktop work, with little direct engagement with frontline health care staff members. This shift requires them to navigate a vastly different environment, requiring new skills, communication styles, and an understanding of clinical challenges. It is not simply a matter of learning new information, it is about adjusting to a completely different way of working, which may be an unwelcome change for people who choose a technical profession.

For collaboration to succeed, stakeholders must reflect on how to adapt their practices, and the additional roles that may be required, to meet the needs of coinnovators and coadopters [30]. However, changing behaviors that are deeply embedded in professional norms is incredibly challenging. There is often an unstated assumption that new theories, models, and approaches can easily be adopted, but research from the fields of knowledge mobilization and translation challenges this view. People are not passive recipients of new ideas; [14] introducing new ways of working involves negotiation, compromise, and integration into established routines [31]. These changes can be disruptive, challenging identities, roles, and responsibilities [20]. Ecosystem members are highly skilled professionals with a large degree of autonomy to choose whether and how to engage with new ways of working [5,15]. Therefore, change requires as much attention to the human side of change, psychological, social, and political, as to the technical side [32].

## Recommendations for How to Address the Challenge: Toward Ecosystem Cohesion

### Overview

To address the fragmentation of health care ecosystems and systemic barriers to collaboration, we propose a set of actionable strategies to work toward ecosystem cohesion. These include adopting a wide-lens perspective to overcome silos, developing a shared ecosystem value proposition to align objectives, promoting ecosystem leadership to enhance collective accountability, encouraging local ownership of ecosystem assessment and improvement, and establishing structured pathways for continuous evaluation and enhancement.

While alternative approaches may also contribute to ecosystem cohesion, our recommendations are comprehensive and well-founded, grounded in empirical research and practical insights. They systematically address core challenges such as fragmentation, misalignment, and inefficiency. By integrating strategic, structural, and leadership-driven solutions, our recommendations provide a robust and sustainable path forward.

### Recommendation #1 Adopt a Wide-Lens Perspective

Building on Adner’s research [5] we recommend that a *wide-lens perspective* is adopted. As Adner demonstrates it is not sufficient to focus on technological excellence at the expense of understanding the needs, experiences, and expectations of innovation adopters and other ecosystem members. There is a need to look over the fence to understand what else is going in the ecosystem that the success of any technological development is dependent on and to understand the implications of this learning for the initial conceptualization and design of new technologies, and the process of their development, testing, and deployment in partnership with other ecosystem members.

These insights suggest there are major limitations to the current “linear” product development mindset, and the metaphor of crossing the “last mile” [11,12]. It is not sufficient simply to attend the final handover of a product between the developers and the intended recipients. The whole ecosystem of coinnovators and coadopters needs to be actively engaged in synchronous and cooperative sense-making and problem-solving [33].

We propose that adopting an alternative process model, such as the cycle for improvement [34] can provide a helpful first step in adopting a wider ecosystem lens. (Figure 1) Rather than the linear progress implied by the innovation pipeline [11], the cycle for improvement emphasizes an iterative, cyclical process that is followed until the desired outcome or improvement has been achieved. The value of such a model is that it provides a holistic view of the entire improvement process and allows practices both upstream and downstream from the point of technology development to be taken into consideration. As such it starts to conceptualize a more holistic and cyclic view of the work required and is likely to appeal to a diverse range of ecosystem members who can see how their work orientates to different aspects of this model.

This process model is only one such example of how the ecosystem can start to be conceptualized in a more holistic manner and is intended as an embryonic starting point from which ecosystem explorations could build. Many other models could also provide valuable starting points such as sociomateriality, which focuses on the intersection of technology, work, and organization [35], and a patient work framework [36] which focuses on the interaction between end users and the innovation. These models operate on the principle that the innovation process is inherently social, integrating human, social, technical, and environmental components [37]. Such models emphasize the necessity of collaboration and coproduction at all stages of the process, rather than having discrete hand-offs between innovators and users that is implied in the linear pipeline model.

### Recommendation #2: Develop a Shared Ecosystem Value Proposition

A major challenge to the health technology industry is that other people in the ecosystem do not share such a technology-centric perspective. Health care’s primary goal is patient care, not the deployment of technology or profit generation, which can create tension and conflict between different stakeholder priorities and incentives.

Adner [5] recommends that the ecosystem should have a *shared value proposition* that reflects the shared aim that all ecosystem members are working toward [33]. The shared value proposition is broader than just the perspective of a focal innovation. It encompasses the entire ecosystem and reflects the interdependencies among all stakeholders focal innovators, coinnovators, and adoption chain partners. While a focal innovation can serve as the starting point for defining the ecosystem it recognizes that every player in the ecosystem must derive value from participating, and their success is interlinked. It reflects a broader focus of the collective ecosystem interests and ambitions, rather than a narrow view that prioritizes the focal innovator’s benefits.

We propose that a shared value proposition could be the aspiration *to improve care with support from the introduction of information-driven technologies*. This statement reflects that any individual technology development is likely to be playing a small part of enabling a wider value proposition that will require many other coordinated actions and innovations to achieve.

The work required to achieve this shared value promotion can be conceptualized as 2 interrelated levels of operation. The first level focuses on *improving care with the support of information-driven technologies*, and the second level focuses on *enhancing the ecosystem’s ability to improve care*. Successful intervention at level 1 (improving care with the support of information-driven technologies) relies on the quality of interaction and relationship between different stakeholders in working together to build a shared understanding of the challenges and potential solutions (level 2) [6].

### Recommendation #3: a Call for Ecosystem Leadership

Ecosystem leaders play an important role in holding a vision for the future potential of the ecosystem, to look beyond individual cases and traditional silos—to consider the ecosystem’s process for innovating and improving patient care works, and how it can be enhanced. This work will include coordinating and streamlining the interaction of ecosystem members, addressing any major gaps, and establishing clear practices, processes, and principles for overall work. It will be important to build on the knowledge and expertise of ecosystem members, while at the same time having the freedom to challenge established norms and ways of working for the benefit of the wider ecosystem. Emphasis should be placed on learning from previous efforts so that mistakes are not repeated, and to establish a forward looking and positive learning culture [38].

While there are substantial benefits to be gained from ecosystem actors working collaboratively to address challenges, there is rarely time or headspace to undertake this work of understanding the ecosystem. As well as potentially contradicting short-term organizational goals, the investment in such boundary work can jeopardize individual career progression if the work conflicts with the expected and rewarded outputs and achievements (eg, financial investment secured each year, number of publications produced, or amount of services delivered). These incentives can be set within an organizational culture, but also often reflect expectations of the professional bodies and recruiters operating across an entire system nationally and globally. This can mean that to participate in boundary work individuals may have to take a personal risk in relation to their job tenure, financial reward, and prospects for internal promotion or career progression with other organizations [39].

Enhancing ecosystem working requires the engagement of system leaders who can remove or replace contrary incentives or create protected spaces where work to strengthen the ecosystem can happen without individuals being penalized [40]. Strategic engagement with system leaders will be important in creating protected spaces for ecosystem work to take place.

### Recommendation #4: Local Ownership of Ecosystem Investigation and Enhancement

In identifying the current system deficiencies and opportunities for change we recognize that this work can only be done by people within the system; it is not something that can be conducted by outsiders and handed over to the ecosystem members to implement [33,41]. People within the system have the necessary insights to understand how things are currently done, what is working well, and what motivations and frustrations they experience. Their engagement is critical to designing and enacting any change ideas, drawing on their foresight of what is likely to work and what challenges may be encountered, in testing and feeding back on the benefits and limitations of any changes in practice, and in their persistence to address and overcome any difficulties experienced.

There is an extensive literature on the features of successful systems change, providing theory, values, principles, strategies, and practical guidance [33]. Much of this work has originated from diverse fields of application such as climate action, education, and social cohesion. While there are many different approaches, frameworks, models, and tools, there is a lot of conceptual and practical commonality between them, suggesting a convergence of thinking from research and practical experience of intervening in complex systems [33,42].

Findings suggest the importance of engaging ecosystem members in the process of investigating and diagnosing the current ecosystem. This supports stakeholders to understand and feel ownership for the identification of problems and areas requiring improvement, increasing their motivation and engagement to explore new ways of working. The engagement of ecosystem members is equally critical in designing, testing, and revising new ways of working to ensure their insights and specialist knowledge is incorporated and to enable the identification and resolution of emergent challenges and problems. No single person or group holds all the knowledge, skills, or competencies required to understand and address all aspects of the changes required. While collaborative working and ownership is view as critical it is recognized as highly challenging given the diverse perspectives and positions different groups and individuals hold, and the need for expert facilitation is recognized to enable constructive conversations between potentially contentious and conflicting views. Therefore, it is not sufficient to simply conduct research to make an objective assessment of the current situation, but necessary to use a participatory action research approach as a tool to support reflection, collective sense-making, and relationship building, in support of the stakeholders working toward system change [43].

### Recommendation #5: a Pathway for Investigating and Enhancing Ecosystems

We propose that it is it helpful to envisage the work required to enhance ecosystems conducted in three stages:

- Stage 1: coinvestigation and diagnosis of current ecosystem functioning
- Stage 2: cocreation of strategies and action plans to address challenges and opportunities to enhance ecosystem functioning
- Stage 3: implementation, evaluation, the evolution of effective strategies and actions—embedding a permanent learning system to guide ecosystem enhancement.

Each of these 3 stages should be informed by bringing together an understanding of specific issues within the local ecosystem and existing knowledge, literature, and priori experiences on ecosystems and intervening in complex systems.

As well as investigating the ecosystem generally, which depending on the ecosystem could be a significant scale challenge—following specific projects developing and introducing information-driven technologies into health care can provide a powerful vehicle to understand ecosystem performance using more bounded and manageable cases (Table 1).

Before an ecosystem can be enhanced it is necessary to understand how the ecosystem is already working. This includes understanding who different members of the ecosystem are, the diversity of approaches and ways of working that they deploy, and how they currently interact with each other.

An important first step in the coinvestigation and diagnosis of the current ecosystem in stage one is to make the ecosystem explicit. This allows people to consider how the outputs of individual, group, and organizational efforts will be adopted by or influence others, and how they in turn are dependent on the efforts and innovations made by other people in the system to improve patient and population health. Research shows that for system change to take place it is necessary to understand what lies below the surface of how people behave [30]. This includes understanding the explicit aspects of the work being done (eg, policies, practices, or resource allocation), those that are semiexplicit (eg, understanding the relationships and power dynamics that inform ecosystem interactions) and implicit (eg, people habits of thought, deeply held beliefs, and assumptions that guide how they work and how they view others in the ecosystem) [44].

The cycle for improvement [24] can be used to guide research and facilitate sense-making about the innovation ecosystem: firstly, in considering what work is being done and why by different ecosystem members relates to the various the cycle stages (1) understand the current situation, (2) identify problems and opportunities, (3) prioritization, (4) identify or develop potential solutions and (5) implementation, and assessing the impact of changes as stage (1) is revisited at the next iteration of the cycle); and secondly in exploring different members and how they work together (who is doing the work and how do they interact together).

In starting to understand the diversity of the ecosystem members, their behaviors, and past experiences, insights will start to emerge as to what is working well, and where problems, frustrations, and challenges are being experienced for the ecosystem members individually and collectively. In some instances, these frustrations may be shared across ecosystem members, and lead to the identification of common goals and agreement to address them. On the other hand, frustrations may identify major gaps or conflicts in how people are working that are not so easily addressed. The latter type of situation are where ecosystem leaders will need to play an important role in either encouraging and incentivizing teams to engage in new ways of working, or by finding other solutions or resources to address the issue.

Suggestions of methods to aid this investigation and diagnosis are outlined in Textbox 1. Once a clear understanding of the current ecosystem has been developed, these methods can also be applied to assess how the ecosystem is changing over time.

In stage 2 stakeholders are engaged in co-design research to establish a shared understanding of the requirements for ecosystem evolution. The objective is to foster a collaborative agreement and commitment among stakeholders regarding strategies, practices, and resource investment to address system issues. Establishing such processes and building practical experience in working together will become increasingly important as more ambitious technologies are developed with greater potential impact and requiring greater collaboration and coordination across diverse ecosystem members. Suggestions of methods to aid this cocreation of strategies and action plans are outlined in Textbox 2.

The implementation of new information-driven technologies within the ecosystem are dealt with in stage three, focusing on achieving growth, learning, and continuous improvement. Suggestions of methods to aid the implementation, evaluation, and evolution of effective strategies and actions are outlined in Textbox 3.

**Table 1:** The use of projects as learning vehicles can be applied at all 3 stages of ecosystem enhancement.

Stage	Purpose of projects as focal point	Action
Coinvestigation and diagnosis	To provide a pragmatic entry point to understand the work taking place within the ecosystem and how different stakeholders are working together.	Review of previous and on-going projects.
Cocreation of strategies and action plans	To provide a platform to explore new ways of working through experimentation.	Using demonstration projects for testing, learning, and iteratively developing ideas until they become workable solutions for ecosystem enhancement.
Implementation, evaluation, and evolution	To further inform ecosystem enhancement and evolution of new and improved ways of working to achieve ecosystem goals.	Systematic learning from projects to increase the scale of application and rigor of evaluation.

**Activities in stage 1: coinvestigation and diagnosis of the current ecosystem.**

Activity and their purpose

**Interviews**

To provide insights into the participant’s unique perspectives of the ecosystem and their underpinning mental models.

**Process and system mapping**

To describe and visualize how the ecosystem is working, understanding who is involved and the roles that they play; how they connect to each other; where there are effective connections, and where there are disconnects. This includes mapping of key activities, actors, connections, disconnects, gaps, and incentives on both structural and organizational level.

**Social network analysis**

To explore the quality and extent of interactions between individuals within the ecosystem and provide baseline data to understand the growth of the ecosystem over time.

**Collaborative workshops and learning events**

To develop a shared understanding of the ecosystem and a collaborative diagnosis of the strengths, weaknesses, tensions, opportunities, and gaps of the current ecosystem.

**Activities in stage 2: cocreation of strategies and action plans.**

Activity and their purpose

**Formation of steering groups**

To establish a focal firm to serve as the central point of coordination for the ecosystem evolution efforts, facilitating collaboration and communication among the various stakeholders.

**Creation of dedicated spaces**

To establish platforms for stakeholders to interact and engage in discussions, knowledge sharing, share insights, collective problem-solving, and test innovative ideas and aims to promote self-reflection, stimulate the generation of creative solutions, and build stronger working relationships.

**Prospective case planning, design, and conduct**

To identify the scope, objectives, and key activities for demonstration projects, developing design principles to guide the implementation of the projects and alignment with the desired outcomes.

**Activities in stage 3: implementation, evaluation, evolution of effective strategies, and actions.**

Activity and their purpose

**Development of ecosystem roadmap**

To guide implementation efforts, with key steps and strategies that are refined and improved based on feedback, insights, and evolving needs.

**Systematic project approach**

To ensure effective execution of projects through the development and application of a systematic project approach.

**Assessment of ecosystem growth**

To gain evidence of the ecosystem’s progress and understand the factors influencing network dynamics through evaluation of; the growth of the ecosystem, dynamics of social networks within it, remaining challenges and opportunities, and effectiveness of network interactions and relationships.

## Conclusion

The successful integration of information-driven technologies into health care systems centers on increasing cohesion and synergistic working between members of the wider innovation ecosystem. This viewpoint provides steps for stakeholders to collaboratively investigate and enhance ecosystem functioning, enabling the effective deployment of information-driven technologies in routine health care practice, to advise the wider ecosystem goal of advancing the quality and efficiency of care delivery. Taking this agenda forward will require strong ecosystem leadership to challenge entrenched norms and behaviors and to engage members in creating new ways of working to enhance ecosystem cohesion and effectiveness.

## Abbreviations

AI: artificial intelligence
NASSS: Non-adoption, abandonment, scale-up, spread, and sustainability
